# Spontaneous spinal subdural hematoma in a geriatric patient under anticoagulant

**DOI:** 10.11604/pamj.2015.21.235.7387

**Published:** 2015-07-31

**Authors:** Samia Frioui, Sonia Jemni

**Affiliations:** 1 Service de Médecine Physique et de Réadaptation Fonctionnelle, CHU Sahloul, Faculté de Médecine Ibn El Jazzar, Sousse, Tunisie

**Keywords:** Spinal subdural haematoma, geriatric patient, anticoagulant, paraparesis, urinary retention

## Image in medicine

Spinal subdural haematoma is rare and may be associated with blood dyscrasia, anticoagulant therapy, lumbar puncture, rupture of arteriovenous malformation, tumour bleeding and spinal trauma. We present a 65-year-old female with history of hypertension and atrial fibrillation. She was on anticoagulant. She presented to the emergency department with an acute lumbar back pain, severe paraparesis and urinary retention. Examination on admission, revealed lower limb paralysis (grade 1/5), cauda equina syndrom and difficulty in self-voiding. A lumbar spine magnetic resonance imaging (MRI) scan was arranged urgently. No coagulation abnormality was detected. MRI demonstrated an epidural haematoma at T12-L1 with spinal cord compression. Decompression was recommended but the patient refused surgery. A complete resolution of the haematoma and neurological recovery ensued without surgical intervention. Neurological recovery was observed within 24 h after Spinal subdural haematoma onset. MRI one month later showed total absorption of haematoma. One year later, the patient always has urinary retention, and she is under intermittent catheterization. Surgical decompression is still the main treatment option of Spinal subdural haematoma, however, a conservative therapeutic approach with careful observation may be considered as a treatment of choice in some cases where surgery is refused (high risk or other reasons) and neurologic recovery is early and sustained.

**Figure 1:**
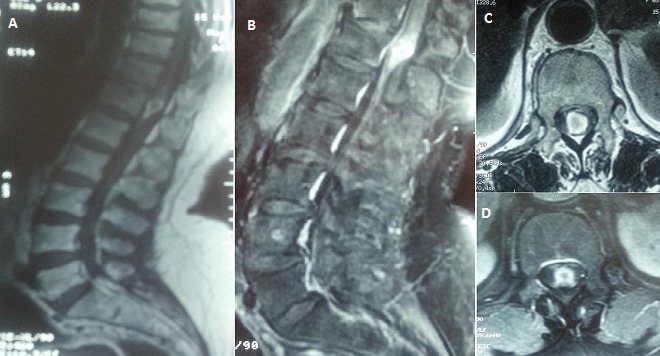
A) sagittal MRI T1 sequence: presence of an epidural haematoma at T12-L1; B) sagittal MRI T2 sequence: epidural haematoma at T12-L1 with spinal cord compression; C) axial MRI T1 sequence: epidural haematoma at T12; D) axial MRI T2 sequence: epidural haematoma at T12, spinal cord compression

